# Supplementary material for: Stretchable Sensors: Novel Human Motion Monitoring Wearables
Source: Nanomaterials (Basel). 2023 Aug 19;13(16):2375. doi: 10.3390/nano13162375 (PMC10459719; doi:10.3390/nano13162375)
Supplement: Supplementary file 1 [file nanomaterials-13-02375-s001.zip › nanomaterials-2559446-supplementary.pdf]

<u>**Supporting Information**</u>

Stretchable Sensors: Novel Human Motion Monitoring Wearables

Chia-Jung Cho*, Ping-Yu Chung, Ying-Wen Tsai, Yu-Tong Yang, Shih-Yu Lin, Pin-Shu Huang

1 Institute of Biotechnology and Chemical Engineering, I-Shou University, Kaohsiung 84001, Taiwan

* Correspondence: ppaul288@isu.edu.tw, Tel: 886-7-6577711*3416 Fax: 886-7-6578945

Table S1. The conductivity of the conductive layer using different fabrication methods.

NO.	Samples	Methodology	Conductivity (S/cm)	Ref.
**1**	Graphene aerogelse	Hummers method/ Freeze thawe	0.17	[1]
**2**	Silver foam	Reduction self-assembly/Freeze dryingt	170	[2]
**3**	Silver nanowire aerogels	Polyol synthesis/ freeze casting thermal annealing	510	[3]
**4**	Silver flakes-polyacrylamide-alginate hydroge	Modified Free radical polymerization/ crosslinking	374	[4]
**5**	Silver nanowire aerogels	Emulsion template synthesist	66	[5]
**6**	polyurethane/PPy (PU/PPy)	Electrospinning/in-situ chemical polymerization	276	[6]
**7**	PEDOT	electrospinning/vapor-phase polymerization	60	[7]
**8**	Silver nanowires in polyvinyl alcohol	Electrospinning/ in-situ AgNPs	650	[8]
**9**	SBS/rGO-AgNPs	Electrospinning/in-situ AgNPs	653	**This work**

References

[1] X. Chen; D. Lai; B. Yuan; M.-L. Fu, *Carbon 162*, (**2020**) 552-561.

[2] X. Wang; W. Guo; H. Zhang; P. Peng, *ACS Appl. Mater. Interfaces 13*, (**2021**) 38637-38646.

[3] F. Qian; P.C. Lan; M.C. Freyman; W. Chen; T. Kou; T.Y. Olson; C. Zhu; M.A. Worsley; E.B. Duoss; C.M. Spadaccini; T. Baumann; T.Y.-J. Han, *Nano Lett. 17,* (**2017**) 7171-7176.

[4] Y. Ohm; C. Pan; M.J. Ford; X. Huang; J. Liao; C. Majidi, *Nat. Electron. 4,* (**2021**) 185-192.

[5] Z. Tian; Y. Zhao; S. Wang; G. Zhou; N. Zhao; C.-P. Wong, *J. Mater. Chem. A 8,* (**2020**) 1724-1730.

[6] S.V. Ebadi; D. Semnani; H. Fashandi; B. Rezaei, *Polym. Adv. Technol. 30,* (**2019**) 2261–2274.

[7] A. Laforgue; L. Robitaille, *Macromolecules 43,* (**2010**) 4194–4200.

[8] K. Yadav; R. Nain; M. Jassal; A.K. Agrawal, *Compos. Sci. Technol. 182,* (**2019**) 107766
